# Deep Learning Based Attenuation Correction of PET/MRI in Pediatric Brain Tumor Patients: Evaluation in a Clinical Setting

**DOI:** 10.3389/fnins.2018.01005

**Published:** 2019-01-07

**Authors:** Claes Nøhr Ladefoged, Lisbeth Marner, Amalie Hindsholm, Ian Law, Liselotte Højgaard, Flemming Littrup Andersen

**Affiliations:** Department of Clinical Physiology, Nuclear Medicine and PET, Rigshospitalet, Copenhagen, Denmark

**Keywords:** pediatric, deep learning, PET/MRI, attenuation correction, brain tumors, bone density, RESOLUTE

## Abstract

**Aim:** Positron emission tomography (PET) imaging is a useful tool for assisting in correct differentiation of tumor progression from reactive changes. O-(2-18F-fluoroethyl)-L-tyrosine (FET)-PET in combination with MRI can add valuable information for clinical decision making. Acquiring FET-PET/MRI simultaneously allows for a one-stop-shop that limits the need for a second sedation or anesthesia as with PET and MRI in sequence. PET/MRI is challenged by lack of a direct measure of photon attenuation. Accepted solutions for attenuation correction (AC) might not be applicable to pediatrics. The aim of this study was to evaluate the use of the subject-specific MR-derived AC method RESOLUTE, modified to a pediatric cohort, against the performance of an MR-AC technique based on deep learning in a pediatric brain tumor cohort.

**Methods:** The modifications to RESOLUTE and the implementation of a deep learning method were performed using 79 pediatric patient examinations. We analyzed the 36 of these with active brain tumor area above 1 mL. We measured background (B), tumor mean and maximal activity (T_MEAN_, T_MAX_), biological tumor volume (BTV), and calculated the clinical metrics T_MEAN_/B and T_MAX_/B.

**Results:** Overall, we found both RESOLUTE and our DeepUTE methodologies to accurately reproduce the CT-AC clinical metrics. Regardless of age, both methods were able to obtain AC maps similar to the CT-AC, albeit with DeepUTE producing the most similar based on both quantitative metrics and visual inspection. In the patient-by-patient analysis DeepUTE was the only technique with all patients inside the predefined acceptable clinical limits. It also had a higher precision with relative %-difference to the reference CT-AC (T_MAX_/B mean: -0.1%, CI: [-0.8%, 0.5%], *p* = 0.54) compared to RESOLUTE (T_MAX_/B mean: 0.3%, CI: [-0.6%, 1.2%], *p* = 0.67) and DIXON-AC (T_MAX_/B mean: 5.9%, CI: [4.5%, 7.4%], *p* < 0.0001).

**Conclusion:** Overall, we found DeepUTE to be the AC method that most robustly reproduced the CT-AC clinical metrics *per se*, closely followed by RESOLUTE modified to pediatric cohorts. The added accuracy due to better noise handling of DeepUTE, ease of use, as well as the improved runtime makes DeepUTE the method of choice for PET/MRI attenuation correction.

## Introduction

Positron emission tomography/Magnetic Resonance Imaging with the combination of MRI and radiolabeled amino acid analog tracers such as O-(2-18F-fluoroethyl)-L-tyrosine (FET) PET offer complimentary information when imaging cerebral brain tumors (Watanabe et al., 1992; Buchmann et al., 2016), especially when estimating the true tumor extent both in low- and high-grade gliomas (Kracht et al., 2004; Vander Borght et al., 2006). The combined information from the two modalities can help to discriminate post-operative changes or radiation damage from true tumor relapse presenting with a contrast-enhanced region (Mullins et al., 2005; Vander Borght et al., 2006; Galldiks et al., 2015a,b). The experience with FET-PET in pediatric and adolescent patients is limited, but it has been shown that FET-PET can add valuable information for clinical decision making (Dunkl et al., 2015). For pediatric patients, there is a clear advantage of acquiring FET-PET simultaneously with conventional MRI, as it offers a one-stop-shop examination, limiting the need for a second sedation or anesthesia as with PET and MRI in sequence, as well as improves co-registration (Henriksen et al., 2016). The advantage of a simultaneous PET/MRI comes with the challenge of accurate attenuation correction (AC) in order for the FET-PET images to be quantitatively correct (Vander Borght et al., 2006).

The initial shortcomings of the vendor-provided AC have been solved for examinations of adult brains without abnormal anatomy to a clinically acceptable precision (Ladefoged et al., 2016), whereas MR-based brain AC methods targeted toward pediatric subjects are scarce. Traditional atlas-based methods are likely to fail, since they are based on a database of adult subjects with normal anatomy (Spick et al., 2016). A database of pediatric age-matched subjects (Bezrukov et al., 2015) is difficult to obtain and might not be sufficient to model anatomical deformations following surgical intervention. An obvious alternative, the MR-based segmentation methods, is often challenged by the fact that traditional MR sequences are not able to distinguish bone and air due to the short relaxation time in bone. However, with special sequences such as ultra-short echo time (UTE) and zero echo time (ZTE), cortical bone can have a high signal despite its very short spin-spin relaxation time (Robson et al., 2003). Unfortunately, the use of these sequences is often hampered by incorrect representation of tissues at air/tissue interfaces (Ladefoged et al., 2015; Sekine et al., 2016) that needs to be specially addressed if a bias is to be avoided. We have recently introduced a PET/MRI-AC method, RESOLUTE (Ladefoged et al., 2015), that makes use of UTE images to calculate an attenuation map with continuous bone representation, and overcomes the air/tissue interface challenges by using anatomical regional masks defined on an aligned template in MNI space. Within these masks, possible bias from the bone surrogate signal is limited. We have shown that RESOLUTE led to the same clinical diagnosis as the reference CT-AC in a challenging cohort consisting of adult post-surgical brain tumor patients with severe anatomical deformations (Ladefoged et al., 2017). A prerequisite for successful application of RESOLUTE to pediatric cohorts is that these masks should be defined on pediatric templates.

Recently, deep learning using convolutional neural networks have demonstrated that they are able to handle complex signals, including noise, while maintaining a high level of accuracy (Han, 2017; Liu et al., 2017; Gong et al., 2018; Leynes et al., 2018). Using this technique, it could therefore be possible to limit the air/tissue interface noise without regional masks, thereby avoiding the need for any registration, as well as benefitting from the improved inference speed usually associated with deep learning. Several techniques using deep learning for MR-AC have been proposed (Han, 2017; Liu et al., 2017; Gong et al., 2018; Leynes et al., 2018), but none have been evaluated on a challenging cohort such as pediatrics.

The aim of this study was to modify the original RESOLUTE method to a pediatric cohort, and implement an MR-AC technique based on deep learning, that takes the UTE images as input and returns an attenuation map without any registration steps or need for regional masks. In a pediatric brain tumor cohort, we evaluated the attenuated FET-PET images of the modified RESOLUTE method, the proposed deep learning method and the vendor-provided DIXON-AC method using CT-AC as reference standard, with the methods evaluated regionally, as well as with metrics used clinically for diagnosis and follow-up examinations.

## Materials and Methods

### Patients

We included children with suspected brain tumor examined with FET-PET using our PET/MRI system (Siemens Biograph mMR, Siemens Healthcare, Erlangen, Germany) (Delso et al., 2011) between February 2015 and October 2017, and 86 FET-PET examinations in total were identified of children under the age of 14. Seven examinations were removed due to missing or corrupt data, resulting in 79 scans used to develop the method (average age: 8 years, min: 2 months, maximum 14 years). For evaluation of the four AC-methods, we included patients with an active tumor area above 1 mL. Patients were part of a larger study of FET-PET/MRI in primary CNS tumors in children and adolescents approved by the regional ethical committee (ID: H-6-2014-095) and registered at clinicaltrials.gov (NCT03402425) and their parents gave written informed consent for participation.

### Acquisition of CT

A reference low-dose CT image (120 kVp, 36 mAs, 74 slices, 0.6 mm × 0.6 mm × 3 mm voxels) of the head using a whole-body PET/CT system was used (Biograph TruePoint 40 and 64, Siemens Healthcare) (Jakoby et al., 2009). The CT images were acquired either on the same day as the PET/MRI examination, or at a previous PET/MRI+CT examination with no brain altering surgery in-between. The longest time for any patient between PET/MRI and low dose CT was 8 month.

### Acquisition of MRI

The scan protocol included two vendor-provided AC methods: a two-point DIXON-VIBE AC sequence with repetition time (TR) 2,300 ms, echo time 1 (TE1) 1.23 ms, echo time 2 (TE2) 2.46 ms, flip angle 10°, coronal orientation, 19 s acquisition time, voxel size of 2.6 mm × 2.6 mm × 3.12 mm, and a UTE AC sequence with TR/TE1/TE2 = 11.94/0.07/2.46 ms, a flip angle of 10°, axial orientation, 100 s acquisition time, software version VB20P, field of view (FOV) of 300 mm^2^, reconstructed on 192 × 192 × 192 matrices (1.6 mm × 1.6 mm × 1.6 mm voxels).

### Acquisition of FET-PET

Patients were positioned head first with their arms down on the fully integrated PET/MRI system. Data were acquired for 40 min immediately following injection of 3 MBq/kg (86 ± 37) MBq FET (Langen et al., 2006) over a single bed position of 25.8 cm covering the head and neck. For the purpose of this study, the summed PET data 20–40 min after injection from the PET/MRI acquisition were reconstructed offline (E7tools, Siemens Medical Solutions, Knoxville, TN, United States) using 3D Ordinary Poisson-Ordered Subset Expectation Maximization (OP-OSEM) with 4 iterations, 21 subsets, zoom 2.5 and 5 mm Gaussian post-filtering on 344 × 344 matrices (0.8 × 0.8 × 2 mm^3^ voxels) in line with the clinical protocol used at our institution. For all images, default random, scatter and dead time correction were applied.

### Attenuation Correction Methods

Four methods for AC were applied to the data. First, the CT image was co-registered to the UTE TE2 image, and was used as our gold standard AC reference following conversion of Hounsfield Units as implemented on the Siemens PET/CT system. Second, vendor-provided MR-based attenuation map were derived using the DIXON VIBE sequence (Martinez-Möller et al., 2009). Third, our recently proposed AC method, RESOLUTE, was updated to process the pediatric cohort on two areas: (1) the regional masks were re-drawn on pediatric templates in MNI space (Fonov et al., 2011) spanning the ages: 0–2 m, <1 year, 1–2, 2–4, 4–8, 8–11, and 11–14 years, and (2) the $R_{2}^{*}$-CT bone mapping was calculated for the pediatric patients by the use of a sigmoid fit rather than a polynomial (Juttukonda et al., 2015). RESOLUTE was derived for each pediatric patient, where we used 2-fold cross validation to ensure that the mapping was not performed on the same patients used to recalibrate the mapping. Lastly, we implemented an MR-AC method based on deep learning convolutional neural networks, denoted DeepUTE. The network was based on a modified version of the U-net architecture (Ronneberger et al., 2015; Çiçek et al., 2016), where the max pool operations were replaced with convolutions with stride 2 (Springenberg et al., 2014), and each convolution, initialized using He normal initializer (He et al., 2015), is followed by a batch normalization, a rectified linear unit (ReLU) activation function, and a dropout layer with increasing fraction from 0.1–0.3 in the encoding part, and vice versa in the decoding part of the network (Supplementary Figure 1). The network takes as input 3D volumes consisting of 16 neighboring slices for each of the three UTE images, the echo images and the derived $R_{2}^{*}$-map (16 slices × 192 voxels × 192 voxels × 3 channels), and outputs the corresponding CT slices (16 slices × 192 voxels × 192 voxels × 1 channel). We used the HU-converted co-registered CT image as our target. We trained the 3D-network in Keras (Chollet, 2015) with TensorFlow backend (Abadi et al., 2016) using the Adam optimizer (learning rate = 10^-4^) (Kingma and Ba, 2014), mean-squared-error as loss function, batch size of 2 for 100 epochs. The 35 million parameters that were determined during the training process took 2 days on a Titan V (NVIDIA Corporation, Santa Clara, CA, United States) graphics processing unit. From our cohort of 79 scans, we did a 4-fold cross validation, effectively training 4 networks on approximately 60 scans and evaluation on the remaining. During testing, we predicted the 3D pseudo-CT volumes around each slice, and computed the average voxel value for each of the overlapping volumes.

Since the CT coverage were usually less than the PET/MRI coverage, we added the DIXON-AC attenuation map outside the CT field-of-view. This was also done for the subsequently generated RESOLUTE and DeepUTE attenuation maps to allow for a fair comparison to the reference.

### Image Processing and Analysis

Image processing and analysis were performed similar to our previous analysis of adult post-operative brain tumor FET-PET patients (Ladefoged et al., 2017). First, a background (B) region of interest was delineated in healthy appearing gray and white matter at a level above the insula in the contralateral hemisphere to the tumor. The biological tumor volume (BTV) of FET-PET was measured using a 3D auto-contour using Mirada XD software (Mirada Medical, Oxford, United Kingdom) defining tumor tissue at a threshold above 1.6 of the mean standardized uptake value (SUV) in the background ROI (Floeth et al., 2005) for each AC method separately. Extratumoral areas with high FET uptake, e.g., vascular structures, pineal body and skin, were identified on either the T1w or FET-PET image and removed from evaluation. The delineation was performed by a nuclear medicine specialist experienced in pediatric neurooncology (LM).

We assessed the different AC methods ability to produce accurate FET-PET images on a patient-by-patient basis using the most commonly semi-quantitative clinical metrics in the diagnostic workflow. We measured the biological tumor volume (BTV), mean (T_MEAN_) and max (T_MAX_), and the ratios T_MEAN_/B and T_MAX_/B were calculated. For the BTV we analyzed the tumor contours relative to the CT-AC reference using the Jaccard similarity metric, and a measurement of shape deviations. The calculated ratios were compared to the ratios calculated with the reference CT-AC. These metrics are commonly used as a criterion to identify active tumor tissue from reactive changes. As described previously (Ladefoged et al., 2017), we defined acceptance criteria of < ± 0.05 and 0.1 or 5% for the T_MEAN_/B and T_MAX_/B ratios, respectively, and ± 2mL or 10% for the BTV. These were based on differences in clinical practice that may be considered clinically relevant in identifying biologically active tumor tissue or treatment related change in activity (Piroth et al., 2011). The mix of both an absolute and relative cut-off reflects that larger absolute change is acceptable in large or very active tumors. For each clinical metric we calculated the mean difference, 95% confidence intervals (CI) and limits of agreement on the log-transformed data, as the data was found to have log normal distribution. Exponentiation was applied to these results to express the differences as ratios on the original scale and report them as percentage differences. We corrected for repeated measurements from the repeated examinations (Bland and Altman, 1999).

## Results

**FIGURE 1:**
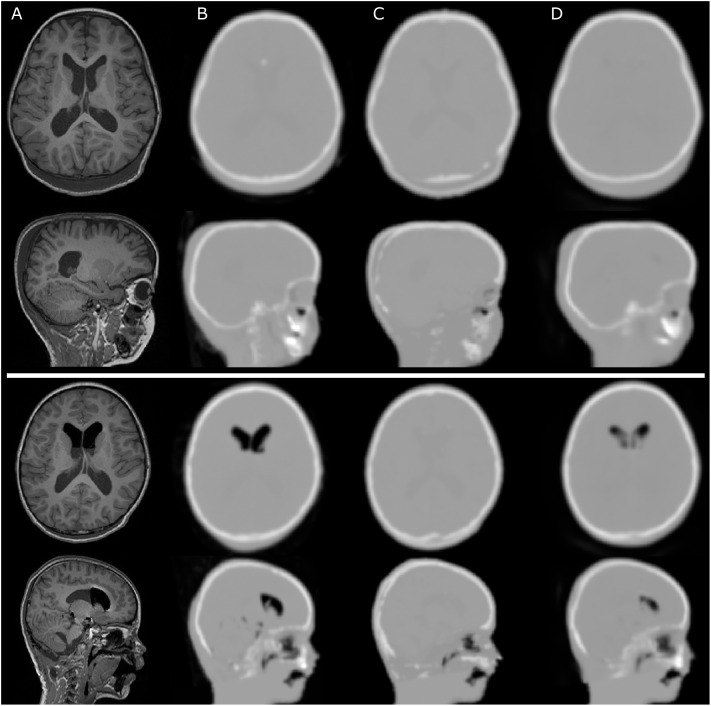
Sample cases for two pediatric patients with irregular anatomy. **(A)** show the T1w MPRAGE, **(B)** CT-AC, **(C)** RESOLUTE-AC, and **(D)** DeepUTE-AC. The top rows show a 5-year-old patient with post-operative subcutaneous soft tissue swelling in the occipital region. RESOLUTE erroneously fills in a dual layer bone layer on both sides of the swelling, along skin and bone. The bottom rows show a 6-year-old patient with air pockets anteriorly in the lateral ventricles that appeared after surgical intervention, and are not imaged in RESOLUTE. Also in this case RESOLUTE crafts a dual layered skull in the occipital region. For both patients, RESOLUTE is challenged in the definition of facial and skull base attenuation value. DeepUTE captures the morphology more confidently.

**FIGURE 2:**
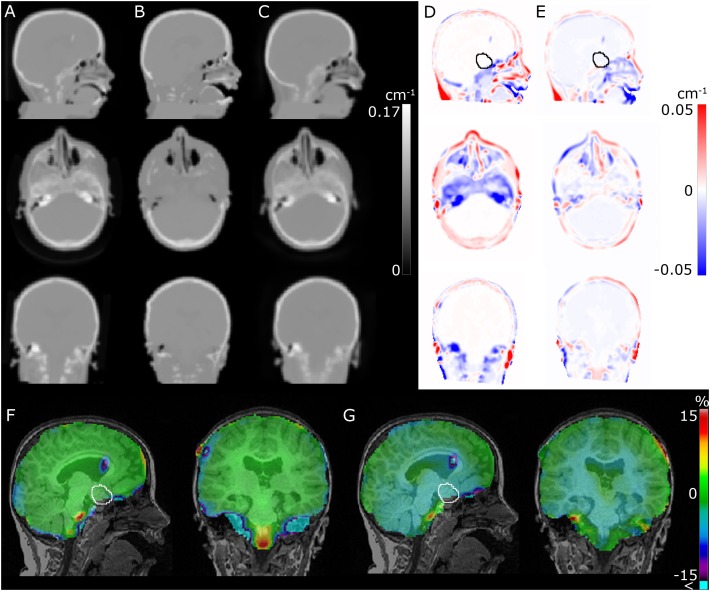
Comparison of CT **(A)**, RESOLUTE **(B)**, and DeepUTE **(C)** attenuation maps in the sagittal, axial and coronal orientation, respectively. **(D,E)** shows **(B,C)** subtracted **(A)**, respectively, and **(F,G)** shows the resulting relative difference in the PET images between RESOLUTE and DeepUTE relative to CT-AC, respectively. The improved accuracy in the nasal cavities, the skull base and the mastoid processes, leads to a clear reduction of the errors in the surrounding regions, e.g., in the medulla. It also appears that, for this patient, a small underestimation of the densities within the brain in DeepUTE leads to a small underestimation globally within the brain. The tumor delineation is show on the sagittal view in **(D–G)**.

A total of 28 patients met the inclusion criteria of 1 mL active tumor area, 6 of which had one or more follow up examination, resulting in a total of 36 examinations used for evaluation (Supplementary Table 1). Both RESOLUTE and DeepUTE were able to derive attenuation maps for all pediatric patients regardless of the age. Ten of the 28 patients (35%) had titanium implants present. Overall, DeepUTE had improved accuracy over RESOLUTE: the Jaccard index was 0.57/0.62 in air, 0.74/0.79 in soft tissue and 0.53/0.70 in bone tissue for RESOLUTE/DeepUTE, respectively. The improved accuracy was also apparent in a direct visual comparison of the estimation of regional attenuation values in the nasal cavities, the skull base and the mastoid processes, and can be appreciated in Figure 1, where two patients with challenging anatomy are shown for RESOLUTE-AC, DeepUTE-AC and CT-AC, and the relative difference PET image in Supplementary Figure 2. Another example of a typical patient is given in Figure 2. There was also a significant improvement in AC runtime with values of 4 s for DeepUTE and ∼3 min for of RESOLUTE, which although small, improves the overall imaging workflow.

Across all pediatric patients, the Jaccard index of the tumor delineation was 0.73 ± 0.20 for DIXON-AC, 0.90 ± 0.07 for RESOLUTE and 0.92 ± 0.07 for DeepUTE. The tumor configuration did not change for any of the patients when using RESOLUTE or DeepUTE compared to CT-AC but for DIXON-AC this was found in 4 examinations (mean difference: 1.6 mL), and was completely missed for an additional examination (BTV with CT-AC: 2 mL).

**FIGURE 3:**
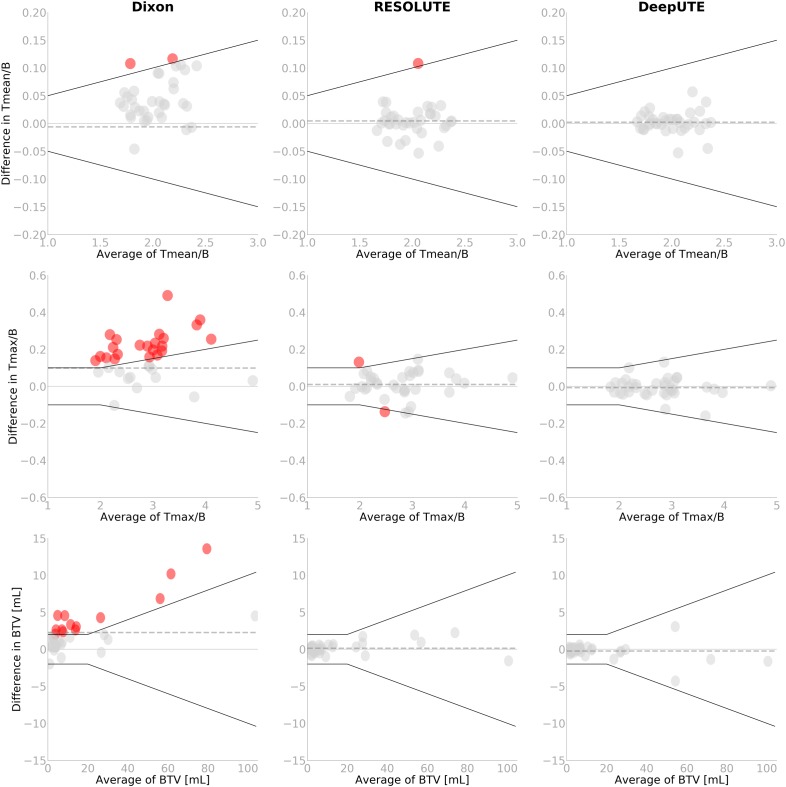
Bland-Altman plot of T_MEAN_/B (top), T_MAX_/B (middle) and BTV (bottom) for the two AC-methods RESOLUTE and DeepUTE against the reference standard CT-AC. The black lines indicate the acceptance criteria of T_MEAN_/B of ± 0.05 or 5%, T_MAX_/B of ± 0.1 or 5%, and BTV of ± 2mL or 10%, respectively. Points that exceed the criteria have been colored. The age of the children exceeding the threshold using RESOLUTE are 7, 7, and 11 years, respectively. Note the difference on the axes. The dashed gray line indicates the mean value.

The comparison of the clinical metrics can be seen in Figure 3, together with the defined acceptable limits. Across all metrics, using DeepUTE, none of the patients were outside the acceptable limits, whereas two patients fall short of the T_MAX_/B limit and a single patient in the T_MEAN_/B limit when using RESOLUTE. In these patients, the largest difference was T_MAX_/B overestimation of 0.13 a.u. due to overestimated bone area in the skull base. In comparison, DIXON-AC gave a T_MAX_/B difference over the acceptable limit in 23/36 (64%) examinations, and 13/36 (36%) examinations had changes to BTV over the acceptable limit.

The relative %-difference in the diagnostic measures was similar between RESOLUTE and DeepUTE, again with DeepUTE with the reduced error and variation (Table 1). BTV measured using DeepUTE was underestimated by 2% on average (95% CI: -5 to 1%) compared to -1% (95% CI: -5 to 4%) with RESOLUTE. None of the metrics had statistically significant differences compared to the reference CT-AC. In comparison, DIXON-AC had statistically significant differences in all three clinical metrics (*p* < 0.001).

**Table 1:** Summary of the relative %-difference^∗^ to the reference CT-AC of each clinical metric for the MR-AC methods.

Measured parameter values	Mean % difference			95% lower limits of agreement	95% upper limits of agreement
			
	Mean	95% CI	*p*		
**DIXON-AC**					
T_MEAN_/B	2.2	1.5 to 2.8	<0.001^**^	-1.6	6.0
T_MAX_/B	5.9	4.5 to 7.4	<0.001^**^	-2.2	14.7
BTV	32	21 to 45	<0.001^**^	-22	124
**RESOLUTE**					
T_MEAN_/B	0.2	-0.3 to 0.7	0.38	-2.6	3.1
T_MAX_/B	0.3	-0.6 to 1.2	0.54	-4.9	5.8
BTV	-1	-5 to 4	0.77	-21	25
**DeepUTE**					
T_MEAN_/B	-0.1	-0.2 to 0.5	0.38	-1.7	2.0
T_MAX_/B	-0.1	-0.8 to 0.5	0.67	-3.7	3.6
BTV	-2	-5 to 1	0.15	-19	17


*^∗^Exponentiation was applied to results from analysis on log scale, and results were expressed as percentages. ^∗∗^Indicates a statistical significant (*p* < 0.05) found by a paired *t*-test. CI = 95% confidence interval for mean difference. BTV is measured in mL. A single examination without BTV with DIXON-AC was left out of this analysis.*

## Discussion

Magnetic resonance imaging is the method of choice to diagnose brain tumor patients, but FET-PET can add valuable information for clinical decision making (Dunkl et al., 2015). Examining pediatric and adolescent patients on a hybrid PET/MRI can be preferred over PET/CT to reduce the number of examinations, which is especially relevant when anesthesia is required, and is important for both child and parents. A prerequisite for a confident clinical evaluation of the cohort with PET/MRI is an accurate AC. The skull shape, density, thickness, and composition change considerably during development in childhood especially the first three years after which the sutures and fontanelles gradually calcify and close (Li et al., 2015). Especially the rapid growth of skull thickness and bone density will highly influence attenuation leading to errors in atlas-based methods that cannot account for the thin, low-density infant cranium.

In designing the clinical study, we were acutely aware of these unresolved AC issues and choose to include a separate low-dose CT acquisition. This could be performed safely in all children, although it involved moving sensitive patients to a different scanner for additional radiation exposure and, for some children, extending anesthesia. This additional stress on the patients was regarded ethically acceptable so that future use of hybrid PET/MRI in pediatric brain tumors, which could be one of the most important applications, could be performed with the best possible assessment of risk to the patient caused by quantitative inaccuracies using accepted standard metrics within the field.

We modified the already thoroughly evaluated RESOLUTE method to be applied on pediatric patients, as well as introduced an MR-AC method based on a deep learning convolutional neural network, and also included DIXON-AC. The novelty of DeepUTE does not lie in the chosen type of architecture, but rather in the data that went into training the model. This manuscript is, to the best of our knowledge, the first of its kind to train a deep learning network for MR-AC purposes on a pediatric cohort of this size. The included patients in the evaluated cohort are well suited to test the method’s ability to adapt to anatomy changes across different ages.

Pediatric patients are a challenging cohort to examine due to motion, often leading to sedation or anesthesia of the patients. The patients included in this study had, as expected, a larger amount of noise in the MR images than adult patients, leading to increased amount of noise in the bone surrogate signal. The strength of the DeepUTE method is that it is able to robustly handle this noise, which the deep learning methods are known for. An example of the improved noise handling is evident in Figure 1, where DeepUTE better models both the thin bone and noise at the posterior part of the head.

Titanium alloy clamps, that were present in 33% of the patients to fix the craniotomy, showed up as small signal voids in the MR images with a size similar to the implants seen on CT. Visual reading showed that both RESOLUTE and DeepUTE filled the signal void with a density similar to dense bone, similar to what has previously been observed (Ladefoged et al., 2017). This meant that a valid attenuation map without artifacts could be calculated in all scans using RESOLUTE and DeepUTE.

Overall, we found both RESOLUTE and our DeepUTE methodology to accurately reproduce the CT-AC clinical metrics with similar accuracy as was seen for RESOLUTE when evaluating adult FET-PET brain tumor patients (Ladefoged et al., 2017). Regardless of age, both methods were able to obtain AC maps similar to the CT-AC, albeit with DeepUTE producing the most similar based on both quantitative metrics and visual inspection. In the patient-by-patient analysis, all patients were inside the predefined acceptable clinical limits with DeepUTE, where three patients (7–11 years old) were outside the limits in the T_MAX_/B or T_MEAN_/B metrics when using RESOLUTE (Figure 3). A similar result was obtained with RESOLUTE for the adult FET-PET brain tumor patients (Ladefoged et al., 2017) where 5/68 studies exceeded the predefined limit. The errors from RESOLUTE were due to an overestimation of bone density in known “problem” areas near the skull base, but none of the errors impacted the clinical reading of the images. In comparison, the same patients obtained with DeepUTE-AC had a higher precision in the skull base, leading to more accurate measurements. The confidence interval was narrower when using DeepUTE compared to RESOLUTE (Table 1). This indicates that there is a smaller variation of the errors in DeepUTE compared to RESOLUTE.

The processing in RESOLUTE was the same for all patients, except for the combination of the segmented tissue maps within regional masks, as these are different depending on the patient age. In DeepUTE, the same method was applied regardless of patient age. Further dividing the training patients into smaller groups depending on age might further reduce the variance, but requires more data, as training a deep learning network with too few patients leads to overfitting. We did not apply transfer learning in this study, as it has been shown that training a deep learning network using less than 30 patients is feasible (Han, 2017; Liu et al., 2017; Gong et al., 2018; Kläser et al., 2018; Leynes et al., 2018). However, using transfer learning, e.g., from a larger adult cohort might further improve the results presented here, as the low-level information are to be expected similar between the cohorts.

In software version VB20P on the Siemens mMR, two vendor-provided solutions for AC is available – DIXON-AC and UTE-AC, that both have been used in the published pediatric neuro-oncology PET/MRI literature (Garibotto et al., 2013; Preuss et al., 2014; Fraioli et al., 2015), however, encompassing only 6 and 12 patients, respectively. This small patient sample may reflect hesitation from the clinical community to use PET/MRI routinely in this difficult patient group because of the well-documented systematic underperformance of particularly DIXON-AC (Andersen et al., 2014; Ladefoged et al., 2017), which is also apparent from our study. DIXON-AC was the only vendor-provided method capable of producing attenuation maps for the full pediatric cohort. In four patients, UTE-AC was not able to produce an attenuation map of patients, aged 0–2 years, which is why we chose to exclude UTE-AC from the comparison.

In this study, we only had 6 patients with repeat examinations. We found that the change of T_MEAN_/B, T_MAX_/B and BTV between two examinations with RESOLUTE or DeepUTE were in congruence with the change when measured with CT-AC, as none of the differences were outside the acceptable limit. A larger number of repeat examinations should confirm this.

### Limitations

We did not have pediatric data available after the software upgrade to VE11P, which adds a model-based AC method (Paulus et al., 2015; Koesters et al., 2016), but we speculate that the method would be unsuccessful for the younger pediatric cohort since the method was developed for adults.

Both RESOLUTE and DeepUTE are based on the UTE sequence, so while we expect DeepUTE to be directly transferable to any Siemens mMR, which is the case for RESOLUTE, neither method is able to produce attenuation maps from PET/MRI data from other vendors. The fundamental idea behind DeepUTE is not limited to UTE data, and retraining the network on other MR sequences such as the T1w MPRAGE or ZTE could allow for a multi-vendor method. However, it would require a large pediatric dataset across several vendors to confirm this.

Although, the limits of agreement using RESOLUTE and DeepUTE are encouragingly narrow (Table 1), the number of patients in each age category is still small. Thus, we cannot rule out artifacts caused by other combinations of anatomy and pathology.

## Conclusion

The present study performed on FET-PET/MRI examinations of pediatric patients revealed that both RESOLUTE and our deep learning method DeepUTE are able to robustly produce attenuation maps similar to the reference CT-AC. The clinical metrics were within acceptable limits of the reference CT-AC, making either method suitable for imaging of pediatric brain tumor patients – a cohort that is especially challenging for atlas-based methods. For clinical use of any MR-AC map, however, we recommend visually inspection for artifacts with particular attention to areas close to the skull base, anatomically distorted tissue and metal implants. The added accuracy due to better noise handling of DeepUTE, ease of use without the need for regional masks, as well as the improved runtime makes DeepUTE the method of choice for PET/MRI AC. Further refinement of the deep learning method with age-specific data input is likely to improve the performance.

## Author Contributions

CL designed the method, did the data analysis, and prepared the manuscript. LM and IL designed the method, aided in data analysis, and revised and approved the manuscript. AH, FA, and LH aided in data acquisition, and revised and approved the manuscript.

## Conflict of Interest Statement

The authors declare that the research was conducted in the absence of any commercial or financial relationships that could be construed as a potential conflict of interest.
