# Supplementary material for: Deep Learning Based Attenuation Correction of PET/MRI in Pediatric Brain Tumor Patients: Evaluation in a Clinical Setting
Source: Front Neurosci. 2019 Jan 7;12:1005. doi: 10.3389/fnins.2018.01005 (PMC6330282; doi:10.3389/fnins.2018.01005)
Supplement: Supplementary file 1 [file Data_Sheet_1.docx]

Supplementary Material

Deep learning based attenuation correction of PET/MRI in pediatric brain tumor patients: Evaluation in a clinical setting

Claes Nøhr Ladefoged, Lisbeth Marner, Amalie Hindsholm, Ian Law, Liselotte Højgaard and Flemming Littrup Andersen*

*** Correspondence:** Flemming Littrup Andersen: flemming.andersen@regionh.dk

# Supplementary Figures and Tables

## Supplementary Tables

**SUPPLEMENTARY TABLE 1**: Patient table for patients and lesions studied with 18F-FET PET/MRI. 10/28 patients had a metal fixation inserted.

| **Patient #** | **Scan #** | **Age at scan** | **Sex** | **Histology at initial diagnosis** | **Location of tumor** | **Prior surgery** |
| --- | --- | --- | --- | --- | --- | --- |
| 1 | 1 | 8y 4m | F | Pilocytic astrocytoma, WHO grade I | Central | 7 months* |
|  | 2 | 8y 10m |  |  |  | 13 months* |
| 2 | 3 | 9y | M | Gigantocellular Glioblastoma, WHO grade IV | Frontoparietal | 20 months* |
| 3 | 4 | 7y 2m | M | Pilocytic astrocytoma, WHO grade I | Fossa posterior | None |
|  | 5 | 7y 5m |  |  |  | 3 months |
| 4 | 6 | 2y 4m | M | NF1 associated opticus glioma, WHO grade I | Optic tracts and chiasm | None |
| 5 | 7 | 13y 11m | F | Diffuse midline glioma with H3 K27M mutation, WHO grade IV | Thalamus | Biopsies |
|  | 8 | 14y 3m |  |  |  | Biopsies |
| 6 | 9 | 10y 5m | M | Pilocytic astrocytoma, WHO grade I | Medulla oblongata | 2 days |
|  | 10 | 10y 9m |  |  |  | 3 months |
|  | 11 | 11y |  |  |  | 6 months |
|  | 12 | 11y 10m |  |  |  | 9 months |
| 7 | 13 | 6y 9m | F | Juevenile xanthogranuloma, No WHO grade | Frontal | 5 months* |
| 8 | 14 | 7y 6m | F | Pilocytic astrocytoma, WHO grade I | Fossa posterior | 5 years |
|  | 15 | 8y 1m |  |  |  |  |
| 9 | 16 | 6y 4m | M | Ependymoma, WHO grade II | Fossa posterior | 1 year* |
| 10 | 17 | 7y | M | Pilocytic astrocytoma, WHO grade I | Fossa posterior | 3 years |
| 11 | 18 | 2m | M | Glioblastoma, WHO grade IV | Central | None |
| 12 | 19 | 7y 5m | M | NF1 associated opticus glioma, WHO grade I | Optic tracts and chiasm | None |
| 13 | 20 | 1y 1m | F | AT/RT, WHO grade IV | Central | Biopsies |
| 14 | 21 | 6y 8m | M | Pilocytic astrocytoma, WHO grade I | Optic tracts and chiasm | 6 years |
| 15 | 22 | 3y 4m | M | Pilocytic astrocytoma, WHO grade I | Fossa posterior | None |
| 16 | 23 | 5 | M | Pilocytic astrocytoma, WHO grade I | Optic tracts and chiasm | Biopsies |
| 17 | 24 | 10y 1m | F | Pilocytic astrocytoma, WHO grade I | Fossa posterior | 1 day* |
| 18 | 25 | 8y 10m | F | CNS HGNET MN1, WHO grade IV | Parietal | None |
| 19 | 26 | 5m | F | Pilocytic astrocytoma, WHO grade I | Optic tracts and chiasm | None |
| 20 | 27 | 3y 9m | M | AT/RT, WHO grade IV | Intraventricular | 14 days* |
| 21 | 28 | 10y 11m | M | Pilocytic astrocytoma, WHO grade I | Medulla oblongata | 2 days* |
|  | 29 | 11y 1m |  |  |  | 2 months* |
| 22 | 30 | 8y 11m | F | Pleomorphic xanthoastrocytoma, WHO grade II | Temporal | None |
| 23 | 31 | 5y | M | Pilomyxoid astrocytoma, WHO grade II | Optic tracts and chiasm | Biopsies |
| 24 | 32 | 7y 5m | M | Pilocytic astrocytoma, WHO grade I | Fossa posterior | None |
| 25 | 33 | 1y 9m | M | Pilomyxoid astrocytoma, WHO grade II | Central | 5 months* |
| 26 | 34 | 4y 7m | F | Pilocytic astrocytoma, WHO grade I | Fossa posterior | 1 day* |
| 27 | 35 | 11y 7m | F | AT/RT, WHO grade IV | Central | None |
| 28 | 36 | 4y 3m | M | Ependymoma, WHO grade III | Fossa posterior | 9 months* |

WHO: World Health Organization. AT/RT: atypical teratoid/rhabdoid tumor. NF1: Neurofibromatosis type 1.

* Metal implant with CranioFix^®^ (n=9/28), Lactosorb^®^ (n=1/28), or titanium plate with screws (n=1/28).
