# Supplementary material for: Deep Learning Based Attenuation Correction of PET/MRI in Pediatric Brain Tumor Patients: Evaluation in a Clinical Setting
Source: Front Neurosci. 2019 Jan 7;12:1005. doi: 10.3389/fnins.2018.01005 (PMC6330282; doi:10.3389/fnins.2018.01005)
Supplement: Supplementary file 2 [file Data_Sheet_2.docx]

Supplementary Material

Deep learning based attenuation correction of PET/MRI in pediatric brain tumor patients: Evaluation in a clinical setting

Claes Nøhr Ladefoged, Lisbeth Marner, Amalie Hindsholm, Ian Law, Liselotte Højgaard and Flemming Littrup Andersen*

*** Correspondence:** Flemming Littrup Andersen: flemming.andersen@regionh.dk

# Supplementary Figures and Tables

## Supplementary Figures

**
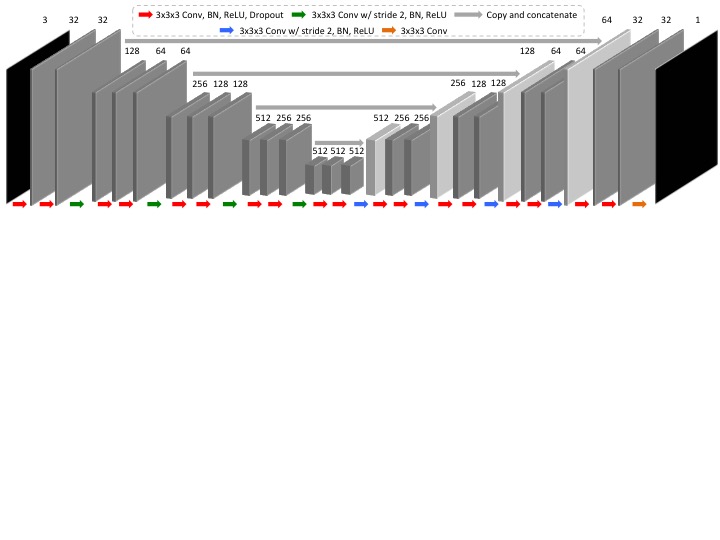
**

**Supplementary Figure 1.** A schematic of the U-net architecture used in this work. The arrows denote computational operations and boxes denote the tensors with the number of channels indicated above each box. The input to the network is the stack of 16 neighboring slices, with the three UTE images echo 1 and echo 2, and the R_2_^*^ map as channels. The output is the predicted DeepUTE image over the same 16 slices.
