# Supplementary material for: Deep Learning Based Attenuation Correction of PET/MRI in Pediatric Brain Tumor Patients: Evaluation in a Clinical Setting
Source: Front Neurosci. 2019 Jan 7;12:1005. doi: 10.3389/fnins.2018.01005 (PMC6330282; doi:10.3389/fnins.2018.01005)
Supplement: Supplementary file 3 [file Data_Sheet_3.docx]

Supplementary Material

Deep learning based attenuation correction of PET/MRI in pediatric brain tumor patients: Evaluation in a clinical setting

Claes Nøhr Ladefoged, Lisbeth Marner, Amalie Hindsholm, Ian Law, Liselotte Højgaard and Flemming Littrup Andersen*

*** Correspondence:** Flemming Littrup Andersen: flemming.andersen@regionh.dk

# Supplementary Figures and Tables

## Supplementary Figures


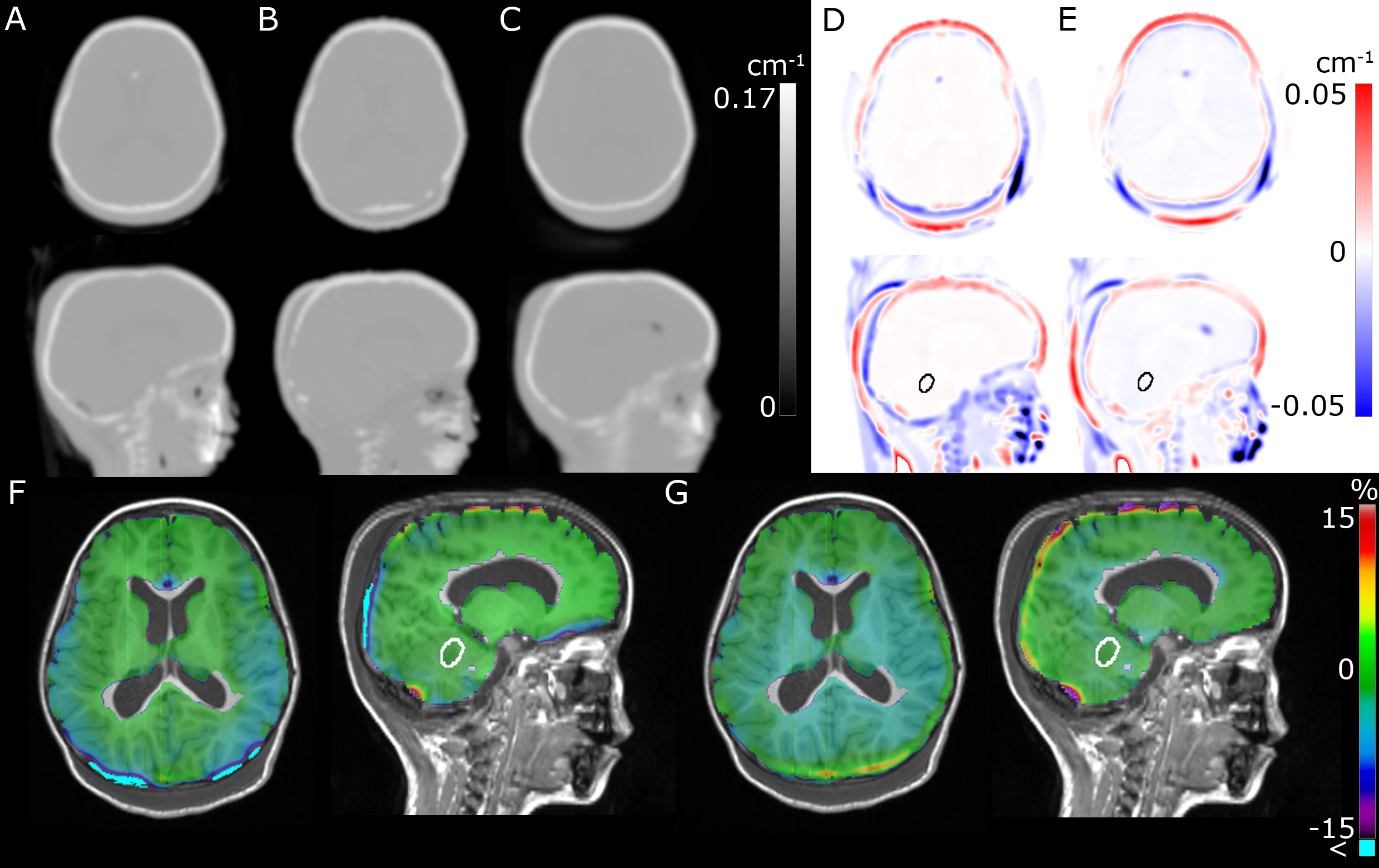


**Supplementary Figure 2.** Comparison of CT (A), RESOLUTE (B) and DeepUTE (C) attenuation maps in the axial and sagittal orientation, respectively. (D-E) shows (B) and (C) subtracted (A), respectively, and (F-G) shows the resulting relative difference in the PET images between RESOLUTE and DeepUTE relative to CT-AC, respectively. The tumor delineation is show on the sagittal view in D-G.
